# Probable Role of Type IV Pili of *Aeromonas hydrophila* in Human Pathogenicity

**DOI:** 10.3390/pathogens13050365

**Published:** 2024-04-28

**Authors:** Agradip Bhattacharyya, Goutam Banerjee, Pritam Chattopadhyay

**Affiliations:** 1 Raja Rammohun Roy Mahavidyalaya, Radhanagar, Nangulpara, Hooghly, West Bengal 712406, India; agradipbhattacharyya@gmail.com; 2 Department of Food Science and Human Nutrition, University of Illinois at Urbana-Champaign, Urbana, IL 61801, USA; 3 M.U.C. Women’s College, Burdwan, Purba-Bardhaman, West Bengal 713104, India

**Keywords:** *Aeromonas hydrophila*, T4P, PIN, operon, synteny, pathogenesis

## Abstract

Background: *Aeromonas hydrophila* is a widely recognized broad-spectrum pathogen that primarily targets the gastrointestinal tract. Type IV pili (T4P) are proteinaceous nano-machines located on the bacterial cell surface, playing a crucial role in host colonization and infection. Regrettably, the T4P systems of *A. hydrophila* remain largely underexplored. Methods: *A. hydrophila* genomes with complete genome assembly and annotation reports up to 31 March 2023, were obtained from the NCBI Genome database or KEGG genome database, followed by a global search for T4P secretion system genes. Protein sequences of these manually curetted genes were used as secondary quarry for Synteny analysis. Protein–protein interaction analysis was performed by string analysis and in silico study of genomic islands. Results: We identified 27 orthologs of type IV pili (T4P) nano-machine components in *A. hydrophila*. These orthologs are primarily distributed across three operons: pilABCD, pilMNOPQ, and pilVWXY. While the first two operons are commonly found in all experimental genomes, the presence of the pilVWXY operon, coding for 11 orthologs, is reported here for the first time in *A. hydrophila*. Notably, the complete pilVWXY operon is absent in nonvirulent strains. A genomic islands study between a nonvirulent and hypervirulent strain also confirms absence of most of the genes coded by pilVWXY in nonvirulent strain. Interestingly, among the 51 experimental genomes analyzed, the pilVWXY operon was completely absent in 10 strains, most of which are categorized as nonvirulent; Conclusions: The distribution of two major type IV pili (T4P) nano-machines, PilABCDMNOPQ and PilVWXY, is reported here for the first time in *A. hydrophila*. Additionally, this study suggests a potential role for the PilVWXY nano-machine in establishing human disease.

## 1. Introduction

*Aeromonas hydrophila* is a ubiquitous, Gram-negative, facultative anaerobic bacterium commonly found in water sources, with a broad host range spanning protozoa [1], mollusks [2], arthropods [3], fish [4], amphibians [5], reptiles [6], birds [7], and mammals [8]. It is known to cause zoonotic infections, transmitted from diseased fish, contaminated water, and partially cooked seafood to humans [9]. These infections can manifest as septic arthritis, gastroenteritis with diarrhea, skin and soft tissue infections, meningitis, and bacteremia [10]. *A. hydrophila* poses a serious threat, particularly to children [11] and immunocompromised individuals [12].

Colonization within the intestinal mucosa is a multistep process facilitated by pili, proteinaceous filamentous appendages found on the surface of many bacteria responsible for host cell adherence. Type IV pili (T4P), a multimeric proteinaceous nano-machine, are expressed by a majority of Gram-negative pathogenic bacteria, including Vibrio cholera, enterotoxigenic *Escherichia coli*, and enteropathogenic *E. coli*. T4P play a critical role in adhesion [13], as they undergo force-driven contraction to enhance attachment to the target surface and facilitate colonization through rapid cycles of extension and retraction, a process known as twitching motility [14]. Additionally, T4P promote surface sensing, motility, biofilm formation, and DNA uptake. They also play a pivotal role in attachment to epithelial cells and GM-1 and GM-2 gangliosides in Pseudomonas aeruginosa [15], facilitating movement across solid surfaces without the involvement of flagella [16]. Mutant strains lacking functional T4P often display impaired attachment and colonization capabilities in *P. aeruginosa* [16,17].

Type IV pili (T4P) in *Aeromonas* are recognized as intestinal colonization factors associated with gastrointestinal infections and diarrhea [18]. *Aeromonas* species possess complete T4P biogenesis gene clusters, such as tapABCD, which are homologous to the T4P gene clusters (pilABCD) [19] found in *Pseudomonas aeruginosa*. Previous studies, such as the work by Boyd et al., have reported the presence of tapMNOPQ in *A. salmonicida* subsp. *salmonicida* [20]. However, limited information is available regarding T4P nano-machine components and their function in *A. hydrophila*. To address this gap, we conducted a comprehensive global search across 51 known strains of *A. hydrophila* using a novel protocol developed for this study. The nomenclature of T4P components or its orthologs across different bacterial genera is not uniform [21]. For example, in *P. aeruginosa* PilV is thought to be part of the tip complex, but in *Neisseria meningitidis,* PilV is an unrelated protein which is incorporated throughout the length of the pilus. Similarly, PilX is a putative tip adhesin in *P. aeruginosa*, homologous to PilK in *N. meningitidis* [21]. To resolve this problem, we used gene ortholog databases (KEGG-KO) to identify the orthologs of T4P components unanimously. To avoid any confusion related to the nomenclature of these orthologs, we use the nomenclature from *Pseudomonas* T4P throughout the manuscript. This protocol integrates KEGG-KO, genome, and proteome databases to investigate T4P components and their potential functions in *A. hydrophila*.

## 2. Materials and Methods

### 2.1. Data Mining

*A. hydrophila* genomes with complete genome assembly and annotation reports up to 31 March 2023, were obtained from the NCBI Genome database (https://www.ncbi.nlm.nih.gov/genome/browse/#!/prokaryotes/1422/) or from the KEGG genome database (https://www.genome.jp/dbget-bin/www_bfind_sub?mode=bfind&max_hit=1000&dbkey=genome&keywords=Aeromonas+hydrophila). This dataset comprises 51 genomes (refer to Table 1). Sequences of stress-responsive enzymes and stress regulators were retrieved from the UniProt database (https://www.uniprot.org/), while protein structures were obtained from the RCSB PDB database (https://www.rcsb.org/). All these databases were accessed on or before 31 March 2023.

### 2.2. Searching for T4P Secretion System Genes

**Figure 1:**
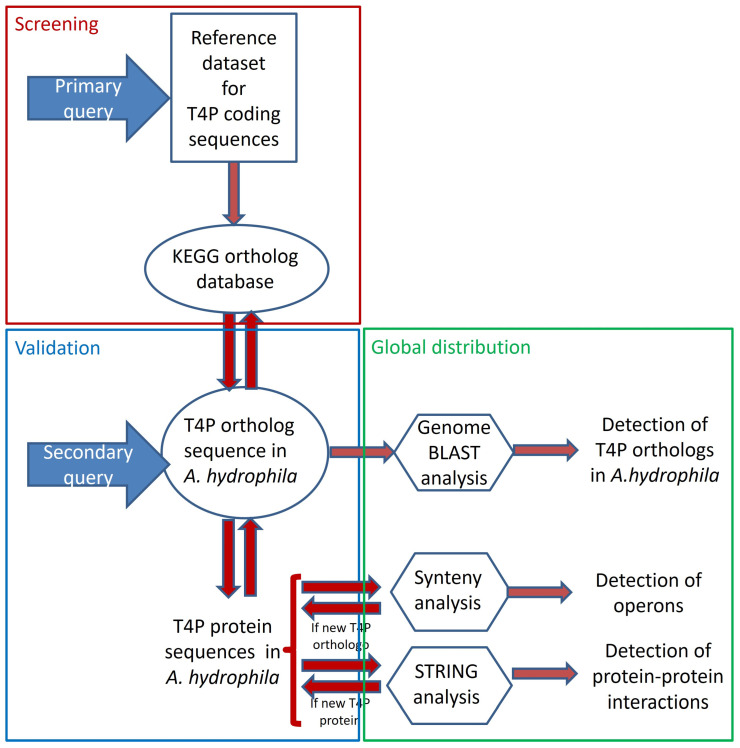
Detailed experimental design and step-by-step workflow.

In this study, we developed a protocol aimed at maximizing the identification of candidate genes related to type IV pili (T4P) secretion systems (see Figure 1). Information regarding the components of T4P secretion systems was gathered from original data previously published by various researchers to create a reference dataset, which served as the primary resource (refer to Table S1). The protein sequences of these manually curated genes were then used as the secondary resource for synteny analysis, leading to the discovery of previously unidentified T4P components in *A. hydrophila*. The results were validated using the KEGG-KO database and further confirmed through STRING analysis (see Table S2). To investigate the distribution of these T4P components across the experimental genomes, a genome blast approach was employed (refer to Table S3).

### 2.3. Synteny Analysis

Synteny analysis is crucial for examining the conservation of gene order, especially in bacterial secretion systems [22]. To investigate synteny among bacterial secretion systems across selected *A. hydrophila* genomes, we utilized the SyntTax server (https://archaea.i2bc.paris-saclay.fr/SyntTax/, accessed on 31 March 2023). According to its creators, SyntTax is a web service tailored to harness the vast array of prokaryotic genomes by connecting them through taxonomic relationships [23]. The functional methodology of SyntTax primarily relies on the Absynte algorithm [24].

### 2.4. String Analysis

STRING is one of the best platforms to construct and visualize protein interaction networks (PINs) [25]. The STRING v11.5 (http://string-db.org) (last accessed on 31 March 2023) server is used for predicting interactions among components of the T4P nano-machine. During STRING analysis, T4P components were aligned with *A. hydrophila* subsp. *hydrophila* ATCC 7966. The first aligned protein with an E-value below 1 × 10^−10^ was considered a homologous protein. Then, these homologous proteins and their corresponding interactions were extracted from the whole interaction dataset of the related organism to compose the model organism-based protein–protein interaction sub-network. To obtain high-quality PINs, we considered the interactions with the highest confidence limits (0.9) from STRING.

### 2.5. Comparing Genomic Islands

Genetic islands encode factors that enhance the adaptability and competitiveness of the bacteria within a niche, including virulence factors and other medically or environmentally important adaptations [26]. Here, we conducted a comparative analysis of genomes of two *A. hydrophila* strains using IslandViewer 4 (http://www.pathogenomics.sfu.ca/islandviewer/, accessed on 31 March 2023) to elucidate the presence and absence of specific genes.

### 2.6. Statistical Analyses

The max-score of all T4P secretion system-related genes was recorded and used to generate a data matrix, which was then utilized to create a heat map visualizing the diversity of T4P components across *A. hydrophila* genomes. The heat map was generated using an online tool called Morpheus (https://software.broadinstitute.org/morpheus/), last accessed on 31 March 2023. Additionally, the same data matrix was employed for multivariate analysis, including Principal Component Analysis (PCA) and cluster analysis (CA), using Past v4.13 software (https://www.nhm.uio.no/english/research/resources/past/, accessed on 31 March 2023). Binary scattered plots were presented following PCA analysis. Furthermore, a cluster dendrogram was constructed via PCA using the p-distance method of neighbor joining with 10,000 bootstraps. Lastly, Venn diagrams were created using an online tool provided by Bioinformatics & Evolutionary Genomics (available at: http://bioinformatics.psb.ugent.be/webtools/Venn/), last accessed on 31 March 2023.

## 3. Results

### 3.1. Searching for Components of A. hydrophila T4P System

A new protocol was developed for identifying components of the *A. hydrophila* type IV pili (T4P) system (refer to Figure 1). Initially, 19 query candidates for bacterial T4P secretion system components were identified based on experimental records (Table S1). Additionally, one additional paralog each for FimT, PilE, and PilZ was confirmed from the KEGG KO database (Table S2). The protein sequences of these 22 T4P components were used as a secondary query for synteny analysis across available annotated *A. hydrophila* genomes (Table S3).

During the synteny analysis, three new candidates, PilT, and two paralogs of PilU (totaling 27) were observed. These were initially verified using the KEGG KO database and then re-confirmed using protein–protein interaction network (PIN) analysis. For the reference strain *A. hydrophila* ATCC 7966, the locus tags for these three new components were identified as AHA_0694, AHA_0695, and AHA_0696 (Table S4). Ultimately, employing this protocol, 27 T4P components were identified in all *A. hydrophila* strains (Table 2).

### 3.2. Synteny among A. hydrophila T4P Systems

**Figure 2:**
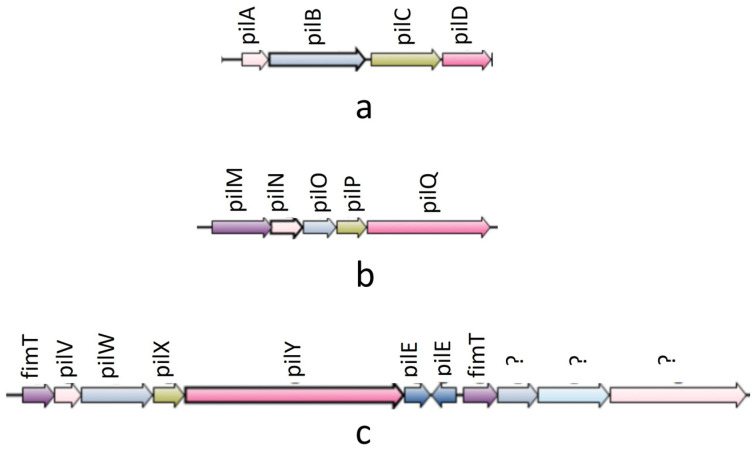
Synteny diagram showing three operons of T4P nano-machine components: (**a**) pilABCD operon: synteny found in all genomes; (**b**) pilMNOPQ: synteny found in all genomes; (**c**) pilVWXY operon(‘?’ marks were put in case of un-identified ortholgs): synteny found in all genomes except Ah2111, AC185, WP7 S18 ESBL 06, AH10, CSUSB2, 71339, MX16A, NEB724, and 4AK4.

In the synteny analysis, 27 T4P components were predominantly found to be organized into three operons: pilABCD; pilMNOPQ; and pilVWXY (see Figure 2). The first two operons were identified in all experimental genomes. Notably, the pilVWXY operon emerged as a major one, encoding 11 T4P orthologs. Interestingly, among the 51 experimental genomes, synteny for the pilVWXY operon components was not observed in 10 genomes, namely Ah2111, AC185, WP7 S18 ESBL 06, AH10, CSUSB2, 71339, A008N2, MX16A, NEB7245, and 4AK4.

### 3.3. Protein–Protein Interaction Network Study

To validate our results, all 27 components (Table 2) underwent a protein–protein interaction (PIN) study within the *A. hydrophila* genome using STRING v11.5. In this analysis, T4P components were aligned with *A. hydrophila* subsp. *hydrophila* ATCC 7966. Proteins with an E-value below 1 × 10^−10^ were considered homologous proteins. Homologous proteins and their corresponding interactions were extracted from the entire interaction dataset of the related organism to construct the model organism-based protein–protein interaction sub-network. To ensure high-quality PINs, interactions with confidence limits above 0.9 were considered from STRING. 

**Figure 3:**
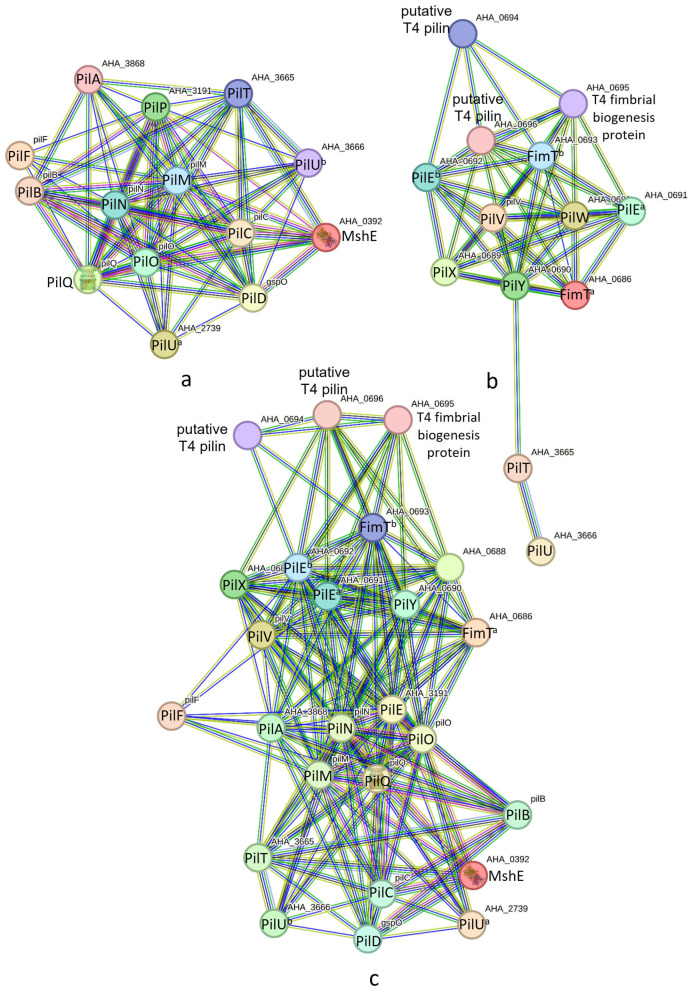
(**a**) Protein interaction networks of PilABCDMNOPQ nano-machine components. (**b**) Protein interaction networks of PilVWXY nano-machine components. (**c**) Protein interaction networks of PilABCDMNOPQVWXY nano-machine components.

T4P nano-machine components encoded in the pilABCD and pilMNOPQ operons were found to interact, forming the PilABCDMNOPQ nano-machine (see Figure 3a). For the PilABCDMNOPQ nano-machine, we identified two separate retraction ATPases encoded by PilT and PilU. These genes were found to be distinct from the pilABCD and pilMNOPQ operons. T4P nano-machine components encoded in the pilVWXY operon exhibited strong PINs (see Figure 3b), comprising 13 nodes (T4P components) and 47 edges (interactions), with an average node degree of 7.23 and a local clustering coefficient of 0.797. In addition to 11 T4P components coded by pilVWXY, two retraction ATPases (PilT and PilU) coded outside the operon may play a crucial role here. The concurrence of PilY and PilT across bacterial genomes may indicate some level of interaction between them (Figure S1). The network within this nano-machine displayed significantly more interactions than expected. Otherwise, T4P components encoded in the pilABCD, pilMNOPQ, and pilVWXY operons all together may form a nano-machine as they exhibited strong PINs (Figure 3c), comprising 25 nodes (T4P components) and 169 edges (interactions), with an average node degree of 13.5 and a local clustering coefficient of 0.8217.

### 3.4. Global Survey of T4P Components within A. hydrophila Genomes

The nucleotide sequences of these 27 T4P components were utilized as tertiary queries for genome BLAST across available complete annotated *A. hydrophila* genomes (see Table S4). In the genome BLAST analysis, pilVWXY operon components did not return any results for the 10 experimental genomes where synteny was also absent (namely Ah2111, AC185, WP7 S18 ESBL 06, AH10, CSUSB2, 71339, A008N2, MX16A, NEB7245, and 4AK4). The differential distribution of the 27 T4P components in *A. hydrophila* genomes was illustrated as a heat map (refer to Figure S2).

**Figure 4:**
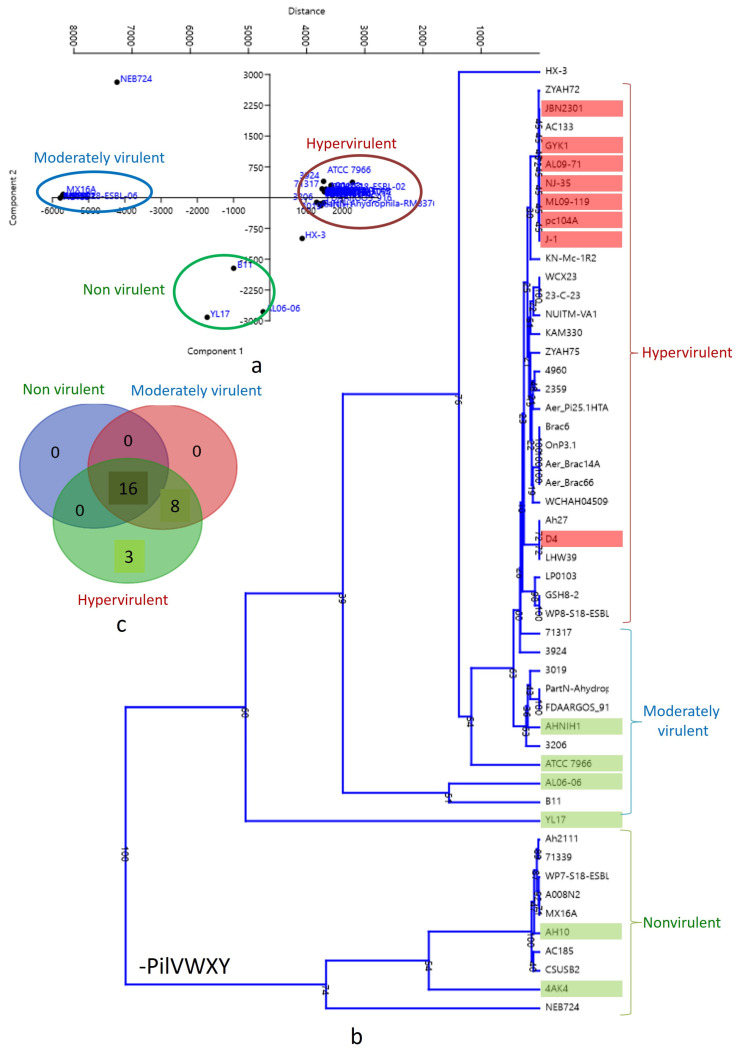
(**a**) Biplot with strains distributed into three clusters by PCA 1 and PCA 2: nonvirulent strains: lower left quadrant; low-virulent strains: upper left quadrant; hypervirulent strains: middle (just above and beneath Y axis) of the right quadrant. (**b**) Dendrogram showing hypervirulent, low-virulent, and nonvirulent *Aeromonas hydrophila* strains in three different clusters. (**c**) Venn diagram showing the correlation of virulence and T4P components in *Aeromonas hydrophila*.

A biplot with PCA 1 and PCA 2 categorized the strains into three clusters (Figure 4a). The lower left quadrant represents nonvirulent strains, while the upper left quadrant represents low-virulent strains. Hypervirulent strains, mostly human pathogens, were clustered in the middle (just above and beneath the Y axis) of the right quadrant. A similar pattern was observed in the dendrogram analysis (Figure 4b), where the 10 strains lacking the pilVWXY operon clustered together at the bottom of the tree. The correlation between virulence and T4P components was illustrated through a Venn diagram (Figure 4c). According to the present analysis, the number of common T4P orthologs among nonvirulent, moderately virulent, and hypervirulent clusters is 16, indicating core T4P components (Table 2). Non-core T4P orthologs were primarily encoded by the pilVWXY operon only. The moderately virulent and hypervirulent clusters shared eight T4P orthologs. The hypervirulent cluster, mainly representing human pathogens, exhibited three unique T4P orthologs, including PilV and two unidentified components (Table 2).

**Figure 5:**
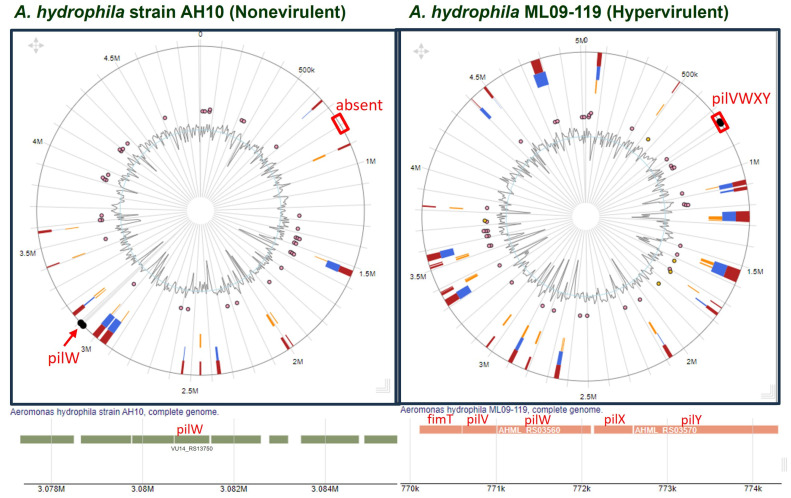
Comparison of genomic islands between a nonvirulent (*A. hydrophila* AH10) and a hypervirulent (*A. hydrophila* ML09-119) strain with special emphasis on pilVWXY.

Synteny analysis indicated the absence of the PilVWXY operon in nonvirulent strains. However, due to recombination, these genes might have changed their position. Thus, to confirm, we compared the genomic region of a nonvirulent strain (*A. hydrophila* AH10) and that of a hypervirulent strain (*A. hydrophila* ML09-119) using IslandViewer 4. Interestingly, the result of the comparative study made it clear that PilV and 10 other components (except PilW) coded by pilVWXY were absent from the nonvirulent strain (Figure 5).

## 4. Discussion

### 4.1. A. hydrophila as a Model

As a pathogen, *A. hydrophila* offers a unique opportunity to study bacterial infections across various animal models due to its broad host range, spanning from protozoa to mammals. *A. hydrophila* has already been proposed as a model for studying infections [1]. The genomes of several strains are readily available in public databases [27]. In our exploration of a specific secretion system, we observed significant divergence across bacterial species, potentially contributing to virulence (Figure 4c). Therefore, this bacterial species has already demonstrated its potential as a model for studying bacterial infections and the underlying molecular machinery.

### 4.2. Emerging New Protocol to Study the T4P Secretion System

There are various standalone programs and server-based applications available for determining type IV pili (T4P), including TXSScan by Abby et al., 2016 [28]. TXSScan identified only one T4P profile in *A. hydrophila* ATCC 7966 and *A. hydrophila* ML09-119 [28]. However, in the present study, we identified 25 T4P components distributed into three operons for both strains. In our previous study, we developed a protocol for identifying all major secretion systems of *Bradyrhizobium* spp., which resulted in better resolution than the standalone program TXSScan [22]. However, type II secretion (T2S) systems are very similar to T4P systems, including all components except the retraction ATPase, and, in some cases, components are shared between the two [29]. For example, *Acinetobacter baumannii* has both T4P and TIIS and only one prepilin peptidase. Therefore, specific protocols for mining specialized systems are crucial. In the present investigation, we identified two potential retraction ATPases (PilU and PilT) for the PilABCDMNOPQ nano-machine. However, the same retraction ATPases may also be shared by the PilVWXY nano-machine. We believe that the present protocol will aid researchers in better mining T4P components in other bacterial systems as well.

### 4.3. Discovery of a New T4P System in A. hydrophila

This marks the first report on the distribution of pilVWXY in *A. hydrophila*. PilV is recognized for promoting bacterial adhesion to human epithelial cells [30], directly interacting with the beta-adrenergic receptor of human endothelial cells [31]. PilV-mediated inter-bacterial adhesion has also been documented [32]. Hence, the presence of PilV may indeed be a determining factor in the virulence of *A. hydrophila* (Figure 4c). In the Venn diagram, it is evident that PilV is a unique T4P component found exclusively in the hypervirulent cluster of *A. hydrophila* (Figure 4c). PilW-mediated active electron transfer has also been proposed for *Acidithiobacillus ferrooxidans* [33]. PilX was detected via immuno-electron microscopy and was associated with type IV pili [34]. Recent evidence suggests that PilX has a global effect on the conformation of pili, implying a potential indirect impact on pilus function [35].

The PilABCDMNOPQ nano-machine (Figure 3a) may adequately fit into the T4P architecture depicted by Craig et al. for *P. aeruginosa* [21]. However, there is also a probability that the T4P nano-machine components encoded in the pilVWXY operon may contribute to the development of a new nano-machine (Figure 3b). This nano-machine may adequately fit into the T4P assembly model proposed by Nguyen et al. for *P. aeruginosa* [36]. There is also a probability that the pilVWXY nano-machine may interact with the PilABCDMNOPQ nano-machine to form PilABCDMNOPQVWXY. This probability is supported by the schematic representation of T4P in *Xylella fastidiosa* by Merfa et al. [37].

### 4.4. Contribution of pilVWXY to Virulence

Based on T4P components, all the experimental genomes were clustered into three groups, i.e., hypervirulent, low-virulent, and nonvirulent, as outlined in the PCA (Figure 4a) and dendrogram analyses (Figure 4b). However, the absence of pilVWXY is the distinguishing feature of the nonvirulent cluster (Figure 4a,c). *A. hydrophila* AH10 and 4AK4 were already reported as nonvirulent strains by Abdella et al. [28]. Therefore, the nonvirulent cluster is well supported by our investigation and by the experimental data of previous workers. A whole-genome comparison of *A. hydrophila* AH10 with *A. hydrophila* ML09-119 (hypervirulent) clearly demonstrated the absence of all or most T4P components of the PilVWXY nano-machine from the nonvirulent strain of *A. hydrophila* (Figure 5).

Though *A. hydrophila* AHNIH1, ATCC 7966, AL0606, and YL17 were reported as nonvirulent by Abdella et al. [28], these strains were reported to cause infection by other researchers. As for example *A. hydrophila* AHNIH1 was reportedly isolated from an infected patient by Rodrigues et al. [38]. Similarly, *A. hydrophila* ATCC 7966 infection in *Carassius auratus* was reported by Peng et al. [39]. Therefore, these strains may be re-classified as low-virulent strains, as evident in the PCA (Figure 4a) and dendrogram (Figure 4b) in our study. Interestingly, both the low-virulent and hypervirulent clusters share components of pilVWXY (Figure 4c). The primary distinction between these two clusters is the presence of PilV, which is unique to the hypervirulent cluster (Figure 4c) and is mainly composed of human pathogenic strains. The adhesive capability of T4P is attributed to the presence of PilY as an adhesin [40]. PilY in *P. aeruginosa* has been shown to be vital for T4P assembly [41] and functions as a mechanosensor, facilitating surface contact-dependent activation of virulence gene expression [42]. Hence, our investigation strongly suggests the role of the newly described T4P system (pilVWXY) as a virulence determinant in *A. hydrophila*.

## 5. Conclusions

Here, we reported the distribution of 27 T4P components in *A. hydrophila*. Our study also suggests the potential role of the PilVWXY nano-machine in establishing human disease. Interestingly, this nano-machine was completely absent from 10 strains, most of which are considered nonvirulent or non-human pathogens. PilV, along with two other orthologs (bearing the locus tags AHA_0694 and AHA_0696 in *A. hydrophila* ATCC 7966), was found to be unique to hypervirulent or human-pathogenic strains. Hence, there is a strong possibility that PilVWXY serves as an important virulence determinant for *A. hydrophila*, contributing to its pathogenicity in humans.

## Figures and Tables

**Table 1:** *A. hydrophila* genomes with complete genome assembly and annotation report used for this study (accessed till 31 March 2023).

Sl. No.	*A. hydrophila* Strain	Accession Number of Replicons	Country of Isolation	Isolation Source/Host
1	OnP3.1	NZ_CP050851.1	Brazil	Aquaculture system/Nile tilapia (*Oreochromis niloticus*)
2	ATCC 7966	NC_008570.1	USA	Humans
3	ZYAH72	NZ_CP016989.1	China	Crucian carp (*Carassius carassius*)
4	WCX23	NZ_CP038463.1	China	Diarrheal snakes/humans
5	3019	NZ_CP053885.1	USA	Fish/humans
6	JBN2301	NZ_CP013178.1	China	Fish with hemorrhagic septicemia/crucian carp (*Carassius carassius*)
7	23-C-23	NZ_CP038465.1	China	Diarrheal snake/humans
8	Ah27	NZ_CP084581.1	China	Liver/catfish
9	D4	NZ_CP013965.1	China	Diseased fish/Wuchang bream (*Megalobrama amblycephala*)
10	LHW39	NZ_CP050012.1	China	Wuchang bream (*Megalobrama amblycephala*)
11	KAM330	NZ_AP023398.1	Japan	Humans
12	NUITM-VA1	NZ_AP025277.1	Viet Nam	Humans
13	AC133	NZ_CP093309.1	South Korea	Kidneys/crucian carp (*Carassius carassius*)
14	Ah2111	NZ_CP095280.1	China	Ascites/humans
15	LP0103	NZ_CP092906.1	Taiwan	Pond/suckermouth catfish (*Hypostomus plecostomus*)
16	AC185	NZ_CP093308.1	South Korea	Kidneys/American eel (*Anguilla rostrata*)
17	GSH8-2	NZ_AP019193.1	Japan	Waste water/ humans
18	WP8-S18-ESBL-02	NZ_AP022252.1	Japan	Waste water/ humans
19	ZYAH75	NZ_CP016990.1	China	Wound secretion/humans
20	GYK1	NZ_CP016392.1	NA	Mandarin fish (*Siniperca chuatsi*)
21	HX-3	NZ_CP046954.1	China	Yellow croaker (*Larimichthys crocea*)
22	WP7-S18-ESBL-06	NZ_AP022206.1	Japan	Waste water/humans
23	KN-Mc-1R2	NZ_CP027804.1	South Korea	Nutria (*Myocastor coypus*)/humans
24	AH10	NZ_CP011100.1	China	Grass carp (*Ctenopharyngodon idella*)
25	AHNIH1	NZ_CP016380.1	USA	Perirectal swab
26	AL06-06	NZ_CP010947.1	USA	Greensboro, Alabama/human
27	CSUSB2	NZ_CP083944.1	China	American alligator’s water tank
28	4960	NZ_CP053883.1	USA	Chickens/humans
29	Aer_Pi25.1HTAS	NZ_CP045501.1	Brazil	Redtail catfish (*Phractocephalus hemioliopterus*)
30	71339	NZ_CP084352.1	NA	Urine of Homo sapiens/humans
31	A008N2	NZ_CP094267.1	China	Water/humans
32	MX16A	NZ_CP018201.1	China	Water/humans
33	B11	CP053859.1	China	Farmed eel/humans
34	Brac6	NZ_CP050850.1	Brazil	Aquaculture system/ fairy shrimp (*Dendrocephalus brasiliensis*)
35	Aer_Brac14A	NZ_CP045502.1	Brazil	Fairy shrimp (*Dendrocephalus brasiliensis*)
36	Aer_Brac66	NZ_CP045220.1	Brazil	Fairy shrimp (*Dendrocephalus brasiliensis*)
37	PartN-Ahydrophila-RM8376	NZ_CP064382.1	USA	Humans
38	FDAARGOS_916	NZ_CP065651.1	USA	Humans
39	71317	NZ_CP084353.1	China	Blood/humans
40	NEB724	NZ_CP050994.1	USA	Humans
41	WCHAH045096	NZ_CP028568.2	China	Sewage/humans
42	AL09-71	NZ_CP007566.1	USA	Channel catfish disease-affected pond/catfish
43	YL17	NZ_CP007518.2	Malaysia	Compost/humans
44	NJ-35	NZ_CP006870.1	China	Fish
45	ML09-119	NC_021290.1	USA	Channel catfish
46	pc104A	NZ_CP007576.1	USA	Soil of catfish pond/catfish
47	J-1	NZ_CP006883.1	China	Humans
48	4AK4	NZ_CP006579.1	China	Humans
49	2359	CP043324.1	USA	Chicken/humans
50	3206	CP043323.1	USA	Chicken/humans
51	3924	CP053884.1	USA	Chicken/humans

**Table 2:** Orthologs of T4P nano-machine components in *A. hydrophila*.

Sl. No.	Name of the T4P Component	KO No.	Locus Tag in *A. hydrophila* ATCC 7966	Function of the T4P Component	* Representing Cluster			Operon
					HV	MV	NV	
1	MshE	K12276	AHA_0392	type IV pilin, mannose-sensitive hemagglutinin (MSHA) pili biogenesis protein, ATP hydrolysis activity	P	P	P	
2	FimT^a^	K08084	AHA_0686	type IV fimbrial biogenesis protein	P	P	Ab	pilVWXY
3	PilV	K02671	AHA_0687	type IV pilus assembly protein	P	Ab	Ab	
4	PilW	K02672	AHA_0688	type IV pilus assembly protein	P	P	Ab	
5	PilX	K02673	AHA_0689	type IV pilus assembly protein	P	P	Ab	
6	PilY	K02674	AHA_0690	type IV pilus assembly protein	P	P	Ab	
7	PilE^a^	K02655	AHA_0691	type IV pilus assembly protein	P	P	Ab	
8	PilE^b^	K02655	AHA_0692	type IV pilus assembly protein	P	P	Ab	
9	FimT^b^	K08084	AHA_0693	type IV fimbrial biogenesis protein	P	P	Ab	
10	** UI	** NA	AHA_0694	putative type IV pilin	P	Ab	Ab	
11	** UI	** NA	AHA_0695	type IV fimbrial biogenesis protein	P		Ab	
12	** UI	** NA	AHA_0696	putative type IV pilin	P	Ab	Ab	
13	PilZ^a^	** NA	AHA_0877	type IV pilus assembly protein (not a part of nano-machine)	P	P	P	
14	PilF	K02656	AHA_1757	type IV pilus biogenesis/stability protein	P	P	P	
15	*** PilU^a^	K02670	AHA_2739	pilus retraction protein, twitching motility protein, ATP hydrolysis activity	P	P	P	
16	PilQ	K02666	AHA_3190	type IV pilus assembly protein	P	P	P	pilMNOPQ
17	PilP	K02665	AHA_3191	type IV pilus assembly protein	P	P	P	
18	PilO	K02664	AHA_3192	type IV pilus assembly protein	P	P	P	
19	PilN	K02663	AHA_3193	type IV pilus assembly protein	P	P	P	
20	PilM	K02662	AHA_3194	type IV pilus assembly protein	P	P	P	
21	*** PilT	K02669	AHA_3665	pilus retraction protein, twitching motility protein, ATP hydrolysis activity	P	P	P	
22	*** PilU^b^	K02670	AHA_3666	pilus retraction protein, twitching motility protein, ATP hydrolysis activity	P	P	P	
23	PilZ^b^	** NA	AHA_3680	type IV pilus assembly protein (not a part of nano-machine)	P	P	P	
24	PilA	K02650	AHA_3868	type IV pilus assembly protein	P	P	P	pilABCD
25	PilB	K02652	AHA_3869	type IV-A pilus assembly ATPase	P	P	P	
26	PilC	K02653	AHA_3870	type IV pilus assembly protein	P	P	P	
27	PilD	K02654	AHA_3871	prepilin peptidase [EC:3.4.23.43 2.1.1.-]	P	P	P	

* Representing cluster: HV (hypervirulent); MV (moderately virulent); NV (nonvirulent); P (present); Ab (absent); ** UI (unidentified); NA (not available); *** Retraction ATPase: PilU^a^, PilU^b^, and PilT.

## Data Availability

*A. hydrophila* genomes with whole-genome assembly and annotation report up to 31.03.2023 were downloaded from the NCBI Genome database (https://www.ncbi.nlm.nih.gov/genome/browse/#!/prokaryotes/1422/ accessed on 31 March 2023) or from the KEGG genome database (https://www.genome.jp/dbget-bin/www_bfind_sub?mode=bfind&max_hit=1000&dbkey=genome&keywords=Aeromonas+hydrophila accessed on 31 March 2023), which includes 51 genomes (Table 1). Sequences of stress-responsive enzymes and stress regulators were retrieved from the UniPort database (https://www.uniprot.org/ accessed on 31 March 2023). Protein structures were retrieved from the RCSB PDB database (https://www.rcsb.org/ accessed on 31 March 2023).

## Supplementary Materials

The following supporting information can be downloaded at: https://www.mdpi.com/article/10.3390/pathogens13050365/s1, Table S1: Reference dataset for components of T4P secretion system from previous publications used as primary quarry; Table S2: Reference dataset used for identification of orthologs for components of T4P secretion system; Table S3: Reference dataset for protein components of T4P secretion system used as secondary quarry; Table S4: Max score of Genome Blast for T4P components across *A. hydrophila* genomes; Figure S1: Co-occurrence of PilY and PilT across genomes indicating probable interaction between them; Figure S2: Heatmap showing distribution of T4P components within 51 *A. hydrophila* genome [30,34,41,42,43,44,45,46,47,48,49,50,51,52,53,54,55,56,57,58].
