# Supplementary material for: Probable Role of Type IV Pili of Aeromonas hydrophila in Human Pathogenicity
Source: Pathogens. 2024 Apr 28;13(5):365. doi: 10.3390/pathogens13050365 (PMC11124393; doi:10.3390/pathogens13050365)
Supplement: Supplementary file 1 [file pathogens-13-00365-s001.zip › pathogens-2899596-supplementary.pdf]

Probable Role of Type IV Pili of *Aeromonas hydrophila* in Human Pathogenicity

Agradip Bhattacharyya [1], Goutam Banerjee [2,*] and Pritam Chattopadhyay [3,*]

[1] Raja Rammohun Roy Mahavidyalaya, Radhanagar, Nangulpara, Hooghly, West Bengal 712406, India; agradipbhattacharyya@gmail.com

[2] Department of Food Science and Human Nutrition, University of Illinois at Urbana-Champaign,
Urbana, IL 61801, USA

[3] M.U.C. Women's College, Burdwan, Purba-Bardhaman, West Bengal 713104, India

* Correspondence: goutamb@illinois.edu (G.B.); pritam.biotechnol@gmail.com (P.C.)

Table S1: Reference data set for components of T4P secretion system from previous publications used as primary quarry

Sl. No.	Gene	Reference
1	fimT	**[1]**
2	mshE	**[2]**
3	pilA	**[3]**
4	pilB	**[4]**
5	pilC	**[5]**
6	pilD	**[6]**
7	PilE	**[7]**
8	pilF	**[8]**
9	pilM	**[9]**
10	pilN	**[10]**
11	pilO	**[11]**
12	pilP	**[12]**
13	pilQ	**[13]**
14	pilT	**[14]**
15	pilV	**[15]**
16	pilW	**[16]**
17	pilX	**[17]**
18	pilY	**[18]**
19	pilZ	**[19]**

Table S2: Reference data set used for identification of orthologs for components of T4P secretion system

Sl. No.	Gene	Locus tag in *Aeromonas hydrophila* subsp. hydrophila ATCC 7966	Ortholog Identification No (KO No.)
1	fimT	AHA_0686	K08084
2	fimT	AHA_0693	K08084
3	mshE	AHA_0392	K12276
4	pilA	AHA_3868	K02650
5	pilB	AHA_3869	K02652
6	pilC	AHA_3870	K02653
7	pilD	AHA_3871	K02654
8	PilE	AHA_0692	K02655
9	PilE	AHA_0691	K02655
10	pilF	AHA_1757	K02656
11	pilM	AHA_3194	K02662
12	pilN	AHA_3193	K02663
13	pilO	AHA_3192	K02664
14	pilP	AHA_3191	K02665
15	pilQ	AHA_3190	K02666
16	pilT	AHA_2739	K02670
17	pilV	AHA_0687	K02671
18	pilW	AHA_0688	K02672
19	pilX	AHA_0689	K02673
20	pilY	AHA_0690	K02674
21	pilZ	AHA_0877	NA
22	pilZ	AHA_3680	NA

Table S3: Reference data set for protein components of T4P secretion system used as secondary quarry

Sl. No.	Protein	Ortholog Identification No (KO No.)	Uniprot ID	Amino acid sequence
1	fimT	K08084	A0KG43	MFLRSGFSLIELMVTVALISILLTLGVPSFSAILRNMTLTSQANNFVAAINLARSEAIRRNTAVTLSATASNLTQNHWESGWQIWVDRNGNGTLDNGELLRLFPDMGAGTLVSNTSLVRFSGNGFLDGRQQVALVFSLQPPECAQEASRDITITPAGRPSIVETSCI
2	fimT	K08084	A0KG50	MEGRKGRSAGFTLIELMVALSLVALLLTVAIPSYQSLRQDQMVKAATQAVYTDMMLLKSEALKRNRNLQMILFNSGTGNWCYRISIDGSCASCNDTCSSIEGRKGVDASEFPGIILTGTYTESSSQIRPISFSPRRGTLPSGSITVSSSAASMKVVTNNLGRVRTCAVSNLVGEVACN
3	mshE	K12276	A0KF99	MAQPRLKMRLGDLLVQEQIISDDQLQLALQQQRQTGRKLGTTLIDLGFISEVQLLQFLARQLDVPFFDLNNLTIDASAVPLLPEVQARRYRALAVNLTDNKVTVAMSDPADLSALDAIAALLRPREMGLAVAREGQLLEYFDRLYRRTREIESFAEQLHEEYQDAGFELGSSNLGAGDEGEATVAKLLRSLFEDAVQVGASDIHIEPDEKVLRIRQRIDGILHENILSEVRIAQALVLRLKLVAGLDISEKRLPQDGRFNMKVRGRDVDVRMSTMPVQYGESVVMRLLDQSSGILSLTETGMPPEILTRFRRQLKRPHGMILVTGPTGSGKTTTLYGALSELNQASQKIITVEDPVEYRLPRVNQVQVNPKIGLTFSHVLRSTLRQDPDILLVGEMRDNETVEIGLRGAITGHLVLTTLHTNDAVTSALRLIDMGAPGYLVASALRAVVAQRLVRRVCEHCVEEKAPDEGQATWLTVLSGEAPGQHVYHKGRGCQSCNFTGYAGRIGVYELLELDQPMMDSLRRNDAEGFAKAARQHPHYRPLALTALDYARQGITSVDEVLRLAEDLG
4	pilA	K02650	A0KPV6	MKKQSGFTLIELMIVVAIVAILAAIALPAYQTYTLRAKYSEVIAAAGPAKTAVEVCVQSLDVTAIDLTNATAGGSCIDAANSALASAIANANAARIDTDNTKTLAAATGANTVTITVTSGTDFANLTPANPTFVMVGTVEASKQVNWTRDTGTCAAASMC
5	pilB	K02652	A0KPV7	MLTMTSSPNSGLALSLAASSLLSESDSQRYLSQAKAQRKPFVTFLIENEILDSKALADFCELEYGVPLLDLAAFDLAEIPQKYLNQKLIEKHHVLPIYTQGHTLYIAMSDPTNVSALEDFGFSFGLHTEALLVEENKLTTAISKLMESDQDALGMEDIDESEISELEVSDEGSRLDESVNTADDDAPIVKYINKIMMDAIKRGASDLHFEPYETKYRIRFRIDGILHEIATPPVNLANRFSARLKVMARLDIAERRLPQDGRIKLKLSRNKSMDMRVNTLPTMWGEKIVIRLLDSSAARLNIEQLGFDERQKAQYLRALSKPQGMILVTGPTGSGKTVSLYTGLNILNTTEVNISTAEDPVEINLPGVNQVQVNPKAGLTFASALRSFLRQDPDVV

				MVGEIRDLETAEIAIKAAQTGHLVLSTLHTNSAAETLTRMMNMGVPAFNIASSVT<br>LIMAQRLARKLCDHCKAPEVVPEAELLELGFTQQQLAAGLRLFKPVGCKECSGGY<br>KGRVGIYEIMLMSENIAKLIMQGANSLQIAAIAQKEGMRTLRTSGLEKARLGVTSL<br>AEINRVTTN
6	pilC	K02653	A0KPV8	MATLTQKQNAPKKVFAFRWSGVNRKGQKVSGELQADSINTVKAELRKQGVNVT<br>KVSKKSQGLFSKGGAKIKPMDIAVVSRQITTMLSAGVPLVQSLQIIARSHEKAAMR<br>ELMGQIAADVETGTPMSEALRRHPRHFDDLYCDLVEAGEQSGALETIYDRIATYRE<br>KSEALKSKIKKAMFYPTMVILVAIVVTSILLLFVIPQFEDIFKSFGAELPIFTQFVIGISR<br>FMQNWWYVIFGGIALAIFLYVRAWRASQKVKDNTDKFILTIPVVGMILHKAAMA<br>RFARTLSTTFSAGIPLVDALVSAAGASGNYVYRTAVMAIRNEVVAGMQINVAMRT<br>VDLFPDMVIQMVMIGEESGAIDDMLSKVATIFEQEVDDLVDGLTSLLEPLIMVVLG<br>VLVGGMVVAMYLPIFKLGSVIH
7	pilD	K02654	A0KPV9	MLLITDVFHSLPWLYFSLVFLFSLMIGSFLNVVIHRLPIMLEREWQAEYLGYFNPETL<br>PQQEERYNLMVPRSACPHCGHAITAMENIPLLSWLWLKGRCRECQAPISVRYPLV<br>ELLTALLSLVVAATFAPGWGLLAALLLTWVLVALTFIDLDKMLLPDQLTLPLLWG<br>GLLFNLAGGFIPLADAVIGAMAGYLVLWSLYWAFKLLTGKEGMGYGDFKLLAAL<br>GAWLGWQALPIVLLLSSLVGAFIGIGLILLRNHHQNKPIPFGPYLAIAGWIALLWG<br>DTITRWYLTTFL
8	Pile	K02655	A0KG49	MRIKQSGITLLELIVVVTIVAIIASVAYPSFTDGLRKSRRAEALKGLLSMQLKQEEFR<br>VSNTSYSATPSQVGNPTSSYYDFSISGATATNYTLIATSKGAQVGDKSGSTICNTLTL<br>NKADTKTPAACW
9	Pile	K02655	A0KG48	MVRGFSLMELMIAVAVTAILTVIAYPSYNSYMASAKRAEAKAALLEAAQYMERQF<br>TADGNYDGGNLASAGLATLPRDGGTAYYNLALNASGASYTLTAIPTGVMNGDP<br>CGLLTLDQGGQQGVSMASMTAAECW
10	pilF	K02656	A0KJ42	MYSQGMDTRTLIVVAALCALPGCVTETTYAGQNSTQREVGPDLKAAAQTRLDLG<br>IQYLQQGNAEQAKFNLDRALQYDPANPQVYVGFAYFYQQVEDFKAAEESYKKAL<br>AMDPSNADAMNNYGAFLCNRGRFDEAEKAFLQAVSQPNYIKIADTYENAALCA<br>AQNRRNDKASEYYRLALGYNPRNPRLLLDMAELSMKDGKLPDVQAYLARFADV<br>SDENENSLWLRLRLAQAMDKPALLHQFGTELVRQYPTSQQAKRYLANDY
11	pilM	K02662	A0KN34	MDVYMFGLFNKGSLLPLAGIDFGSQTIKAVTITGRPGKLHLESVAEVATPKGTLVD<br>YQLQDIERVSQSLKALKRLISGSSQYVATAVTGSNVITKVIQVDAALGENELENQV<br>QLEAEQLIPFPLDEVSLDFEILGKVNDQERREVLLSAARTESVSGRVTALAEADMTT<br>KVVDVGAHALGRAVLACLPELQEWDKPVGVIDIGASAMTFAALVKGEVIYSRLQ<br>NFGGDQYSQALASFYNLSLDDAEQAKLQGKLPVDHELDVLLPHMNALLQQVRR

				NVQLFCSSSGHRELSRLVLTGGGSLLPGLAAQVGSELNCEVLHPDPFALFGKPKGE<br>GAVHGAKFMTALGLALRSFTPCQI
12	pilN	K02663	A0KN33	MSNINLLPWREARAQRQKKQFGVMLGIFMAITASLGFAADWLVEQQIGHQQQR<br>NQRLQQEMTILDAQLGEIRLLKERRKELIDRMQLIEHLQMRRNLPVRLFNQLPSLV<br>PNGVYLNTLALQNNQIDVNGKTEAYGRVASMMRRIDGSGWLGQSQISTIFAADV<br>APVSLSQFSMMFQVAGAAGATVDVAKGQQ
13	pilO	K02664	A0KN32	MNLQQLNELDLNNIASWPKLAKGIFILFFCALLGGAIYYYVIANSLTLLTQETNKE<br>AELKAQFESKAMLAANLGAYKTQMVQLEQLVDTQLKQLPNTHEVAGLLDDISFI<br>ATDNGLKLNRINWEPEIKHEFSTELPMRIEVVGTYHEIGKFTADMAALPRIVILESF<br>TLGQGKEQGDMIAMSMLAKTYKYNGKTVGQTK
14	pilP	K02665	A0KN31	MKLLCVLLPALLLTACGGQDDMDNYVAATKARKPVPIEPLPEIKPFSPMAYHLSD<br>QRSPFIAPQPETSSAKVDAKVKPDCAQIVANREKEVLERYSLASLSMQGSLGKQGQ<br>LWALIRTPDGQSIRVGLNQHMGLDQGRVIRITDTYVDLIETIPDGKGCWVTRETQL<br>GMANLEAKR
15	pilQ	K02666	A0KN30	MKTTIGMVARVTLLFCTGAWSQAWAVATLQEVKVNPLMADQLLLELSFSEPVSG<br>FTDRLSYEPNQLLLHVPGAVGALNVNPLPIKQQGVDNLKVEGKGAGLDIKIALDQ<br>LTPYQVHQQGNKLLVALGEKAAMPLPATTASSGLVAPQPTSSALINTQPAASSALI<br>NSQQLARQSAPVAASVASKPVLPSPQTAASGAYFNSVKGVDFRRGKDGQGEFLVT<br>LDNSSAAVDVSSRGQTVLAKFHGTRVPDDLLNLINVQDFATPVSQVEVFRQGNDT<br>LFELSVNGQFDYRYDQADKMFIVEVKKRTAATAGKQYQGKPISLNFQDIPVRTVL<br>QLIADFNNLNLVTTDSVSGNITLRLDGVPWEQALDIILKVRGLDKRLDNNILLVAP<br>AEEIAAREKQQLESRNQVADLAPLYTEYLQINYAKASEVAALLSSESTKLLSSKGAV<br>SVDERTNVLVVKDTADVISNIKRMLDILDIPVKQVVIEARMVTIDDGFDEALGVRW<br>GVTKNDGHGNSTSGTIEGNDSSGNNNGGSTITRPGVDDRLNVNLPVTNAAGTLA<br>FQVARLADGTLLDLELSALEKESKAEIIASPRVTTANQKPALIEQGTEIPYVESSSG<br>ATSVTFKKAVLSLKVTPQITPDNRVILDLTVTQDTKGETVPTGTGDAVSINAQSITT<br>QVLVNNGETLVLGGIYQQTIKSDVSKVPLLGDIPGLGVLFRKTTSENKKRELLIFVT<br>PKIVTDAF
16	pilT	K02670	A0KLU6	MELRDMLQILAKQDGSDLYLSTGAPPCAKFNGGLRPLSETPLEPGEVARIADAIMD<br>GEQKQQFERELEMNLAISLPQIGRFRINIFKQRNEVSLVARNIKTEIPRFEDLKLPPV<br>LLDTIMEKRGLVLFVGGTGSGKSTSLAALIDHRNRNSGGHIITIEDPVEFVHRHRKSI<br>INQREVGVDTRSFHAALKNTLRQAPDVILIGEIRDRETMEHALAFSETGHLAISTLH<br>ANNANQALDRIINFFPEERRPQLLNDLGNNLKAFVSQRLVKTSDGGRRAAVEIML<br>GTHTIRDMIKRGEFGGLKEVMEKSKALGMVTFDSALFDLVVEGVIDEEEAVKNAD<br>SANNLRLKIKLWKEKGQIASSSDATGWSLEPTKDEKDDLF

17	pilV	K02671	A0KG44	MKRHQGVSLLEIMIAVLVLSIGILGMATLQLQALKSNQSALTRTEATQFGYMITDM<br>MRANRSAALLGQYNVGLGEAVSGSSMAIQDVQYWKQALTGLPGGDGAIAVSAG<br>QATITIQWNASRLATEPALRSMTLRTDL
18	pilW	K02672	A0KG45	MSGRRTLLRQAGVGLVEVMIALLLSLLTVGVIIQVLLGNHKTYLTGEAIARVQEDS<br>RFAVNLLQQELRMVGYQGCLSKQGVNITNTLNGGTALPYNFTVYLRGYDNVTAT<br>LPTALSALFTAAEPKPKPGTDVLLVQGPTGAGVPVTRNNSAAQLFVQQLSSKANY<br>CGTGKQGYSDLCEGDIVMVSDCQKARIFQITQTQVVGGGSEVNIGHSNDNKYTPG<br>NTVSSWGGASSPVEERFGAGSVLSRMETRIYYIARPSANAPYALYRKSGLAAGMLL<br>VDGVTDVQLTFGEDQNRDRAADRYVSAASNPNWDNVLGIGVQLLMRSGQGNV<br>VSDPQAISFAGATFQAVDNHWYWVAETTVALRNRLP
19	pilX	K02673	A0KG46	MMARQGGMALVISLIFLAVVSLLAMASMQSALLQEKMAGNQKESQQALQAAEA<br>ALRAAERYLEAGSSGPYDNSAGLYEFVSVAVDPASPSTAWRTYANSGLSGRAPEYF<br>IERLPYTQGSNESLAVDEPISERRLYRITARGFGLSDESRVLLQSTYSR
20	pilY	K02674	A0KG47	MRASGYGLSALLLCTQVQAALDIAQVPLYLGTRAEPNIMFSLDDSGSMHFELMPE<br>GLIENSARYVYPRADNVYGSEDYDNRTVTFTSNNDRNAYTRSSNNNKLYYNPQQ<br>SYRPWAKADGTLMANASISCAPHNPFNTAAGCRDLTKNNRSKSLRYYTGFSSTDY<br>NDDETFWPAVYFAYKGSGDVKKVGSYTRVEIKSGSTYGGRPNRSDCKSAPVCTYD<br>EEIQNFANWYTYYRSRILAARAGVGRAFASQGQALRVGFATINASGSVIRGVAPFS<br>GTDRSAFFSDLYSRDIPAAGTPLRTSLKDVGEYFSRTDNNGPWAASAGSTLPHLTC<br>RQSYNILMTDGYWNGDTPSVGDLDKDGYSNTLADVAYKYWKTDLRADLADKVP<br>TSTADPANWQHLVNFTVGLGVNGSLNPANGIPNRWPNPHDPDDKEYKNATYIP<br>EYKIDDLWHAALNSKGSFFSAGDPDIFAAALSSTLAQIAARNSSASSVTANATRLD<br>SNTHIYQARYNSGDWSGQLISIPLNSDGSLGNMAWDAATLIPAHTSRSIFTRQNGV<br>GIPFTWAALNATNRALFNLAGDSQGENRVAYLRGDRSREQSNGGLFRSRSDLLGD<br>IINSDPVYVGSRDYGYGSATGLTQAERDGYLSFLGSTAIRSRTPMLYVGANDGMLH<br>GFRVANGVETLAYIPVSLLGDLSLLTKPDYSHRYYVDGTAKVGDAYLGSSWKTVL<br>LGSTGAGGKAVFAIDVTAPDNFTADKMLWEFTNTEMGVALAAPTLVRVKSGNK<br>WVALVANGYNSTSQTARLFVLDLATGAVIKEIDTQVGSASEPNGLSSPLPVDEDG<br>DRVADYVYAGDLQGNLWKFDLTDNNSAQWGSAFKTGNKPKPLFQACNGTCSA<br>STRQPVTMRPLAIRHPKGGIMVLMGTGSYFTNDDKLLPATPRLEAVYGIWDTGAS<br>VLSSQLLQQSITHEYSANGTTIKFNVRVVSNTGVNYTSQKGWYLVLKSPALSKGVG<br>ERAVSEMLYRNKRLIFNTLIPSADACDFGGRSWL<br>MELDPVSGARLTYSVFDVNGDGAVNDDDYVGIKDSGGNDIKVPVSGKQFDELTT<br>TPSVVEDADMERKYISGSSGNISVTLEEGAGDLGGRQSWLQLE

21	pilZ	NA	AHA0877	MGGGQHNCSCGPARRRQTTEDVRVITSTKERRAFQRMIINAPVTIFQQQQVLEGVCRDLSANGMGIAVAEHQLDVSQPIRVSLATNNNLLPPFEAQARIIRVLEEEDGLLLAIEFQALA
22	pilZ	NA	AHA3680	MSERRRFSRILYLTMADLVQGDKKWRTQLVDISLQGALLIRPDDWESHDNKEYSLSFVLSGSDIEIKMQVMLTHEASKKLGFYCHHIDIDSATHLKRMIELNVGDEDLLHRELEQLLSEHLEHPHP

Table S4: Max score of Genome Blast for T4P components across *A. hydrophila* genomes

Sl. No.	T4P Component	KO No.	Locus tag	**OnP3.1**	**ATCC 7966**	**ZYAH72**	**WCX23**	**3019**	**JBN2301**	**23-C-23**	**Ah27**	**D4**	**LHW39**
1	MshE	K12276	AHA_0392	2931	3158	2887	2959	2920	2898	2959	2898	2898	2898
2	FimT	K08084	AHA_0686	898	931	881	898	898	881	898	881	881	881
3	PilV	K02671	AHA_0687	688	765	704	699	699	704	699	477	477	477
4	PilW	K02672	AHA_0688	1901	2023	1840	1868	1901	1840	1868	1840	1840	1840
5	PilX	K02673	AHA_0689	826	881	848	854	852	848	854	848	848	848
6	PilY	K02674	AHA_0690	5707	6194	5712	5674	5740	5712	5674	5712	5712	5712
7	PilE	K02655	AHA_0691	688	743	693	710	676	693	710	693	693	693
8	PilE	K02655	AHA_0692	604	715	643	693	610	643	693	643	643	643
9	FimT	K08084	AHA_0693	865	992	881	953	920	881	953	881	881	881
10	NA	NA	AHA_0694	953	1153	837	976	1014	837	976	837	837	837
11	NA	NA	AHA_0695	688	743	693	710	676	693	710	693	693	693
12	NA	NA	AHA_0696	3386	3878	3419	3441	3003	3419	3441	3419	3419	3419
13	PilZ	NA	AHA_0877	688	743	693	710	676	693	710	693	693	693
14	PilF	K02656	AHA_1757	688	743	693	710	676	693	710	693	693	693
15	PilU	K02670	AHA_2739	1929	2106	1917	1945	1984	1917	1945	1917	1917	1917
16	PilQ	K02666	AHA_3190	688	743	693	710	676	693	710	693	693	693
17	PilP	K02665	AHA_3191	688	743	693	710	676	693	710	693	693	693
18	PilO	K02664	AHA_3192	688	743	693	710	676	693	710	693	693	693
19	PilN	K02663	AHA_3193	688	743	693	710	676	693	710	693	693	693
20	PilM	K02662	AHA_3194	688	743	693	710	676	693	710	693	693	693
21	PilT	K02669	AHA_3665	1929	2106	1917	1945	1984	1917	1945	1917	1917	1917
22	PilU	K02670	AHA_3666	1924	2101	1912	1940	1979	1912	1940	1912	1912	1912
23	PilZ	NA	AHA_3680	688	743	693	710	676	693	710	693	693	693
24	PilA	K02650	AHA_3868	132	893	171	159	169	171	159	171	171	172
25	PilB	K02652	AHA_3869	688	743	693	710	676	693	710	693	693	693
26	PilC	K02653	AHA_3870	2061	2294	1995	2100	2100	1995	2100	1995	1995	1995
27	PilD	K02654	AHA_3871	1463	1613	1408	1441	1447	1408	1441	1408	1408	1408

Table S4: (Contd.)

Sl. No.	T4P Component	KO No.	Locus tag	**KAM330**	**NUITM-VA1**	**AC133**	**Ah2111**	**LP0103**	**AC185**	**GSH8-2**	**WP8-S18-ESBL-02**	**ZYAH75**	**GYK1**
1	MshE	K12276	AHA_0392	2926	2959	2898	2998	2964	2904	2909	2909	2915	2898
2	FimT	K08084	AHA_0686	887	881	881	0	887	0	909	909	876	881
3	PilV	K02671	AHA_0687	688	682	704	0	665	0	682	682	682	704
4	PilW	K02672	AHA_0688	1884	1823	1840	0	1862	0	1879	1879	1890	1840
5	PilX	K02673	AHA_0689	832	848	848	0	848	0	843	843	815	848
6	PilY	K02674	AHA_0690	5790	5696	5712	0	5768	0	5768	5768	5840	5712
7	PilE	K02655	AHA_0691	710	699	693	0	660	0	660	660	688	693
8	PilE	K02655	AHA_0692	688	643	643	0	693	0	693	693	627	643
9	FimT	K08084	AHA_0693	953	959	881	0	931	0	931	931	887	881
10	NA	NA	AHA_0694	942	959	837	0	1020	0	1020	1020	992	837
11	NA	NA	AHA_0695	710	699	693	0	660	0	660	660	688	693
12	NA	NA	AHA_0696	3496	3430	3419	0	3602	0	3602	3602	3413	3419
13	PilZ	NA	AHA_0877	710	699	693	693	660	693	660	660	688	693
14	PilF	K02656	AHA_1757	710	699	693	693	660	693	660	660	688	693
15	PilU	K02670	AHA_2739	1967	1923	1917	1912	1962	1912	1940	1940	1956	1917
16	PilQ	K02666	AHA_3190	710	699	693	693	660	693	660	660	688	693
17	PilP	K02665	AHA_3191	710	699	693	693	660	693	660	660	688	693
18	PilO	K02664	AHA_3192	710	699	693	693	660	693	660	660	688	693
19	PilN	K02663	AHA_3193	710	699	693	693	660	693	660	660	688	693
20	PilM	K02662	AHA_3194	710	699	693	693	660	693	660	660	688	693
21	PilT	K02669	AHA_3665	1967	1923	1917	1912	1962	1912	1940	1940	1956	1917
22	PilU	K02670	AHA_3666	1962	1918	1912	1907	1957	1907	1935	1935	1951	1912
23	PilZ	NA	AHA_3680	710	699	693	693	660	693	660	660	688	693
24	PilA	K02650	AHA_3868	161	171	171	182	165	132	137	137	126	171
25	PilB	K02652	AHA_3869	710	699	693	693	660	693	660	660	688	693
26	PilC	K02653	AHA_3870	2061	2078	1995	2073	2073	2084	2067	2067	2100	1995
27	PilD	K02654	AHA_3871	1441	1480	1408	1430	1441	1530	1435	1435	1430	1408

Table S4: (Contd.)

Sl. No.	T4P Component	KO No.	Locus tag	**HX-3**	**WP7-S18-ESBL-06**	**KN-Mc-1R2**	**AH10**	**AHNIH1**	**AL06-06**	**CSUSB2**	**4960**	**Aer_Pi25.1HTAS**	**71339**
1	MshE	K12276	AHA_0392	2920	2998	2937	2942	2953	2987	2915	2920	2920	2998
2	FimT	K08084	AHA_0686	893	0	887	0	893	909	0	887	898	0
3	PilV	K02671	AHA_0687	676	0	671	0	682	660	0	688	665	0
4	PilW	K02672	AHA_0688	1851	0	1857	0	1857	1845	0	1834	1829	0
5	PilX	K02673	AHA_0689	848	0	854	0	859	843	0	832	837	0
6	PilY	K02674	AHA_0690	5690	0	5773	0	5729	5651	0	5738	5712	0
7	PilE	K02655	AHA_0691	660	0	676	0	627	665	0	676	693	0
8	PilE	K02655	AHA_0692	627	0	616	0	599	555	0	638	621	0
9	FimT	K08084	AHA_0693	893	0	893	0	870	870	0	881	909	0
10	NA	NA	AHA_0694	848	0	843	0	965	0	0	965	992	0
11	NA	NA	AHA_0695	660	0	676	0	627	665	0	676	693	0
12	NA	NA	AHA_0696	2047	0	3413	0	3059	0	0	3413	3419	0
13	PilZ	NA	AHA_0877	660	693	676	693	627	665	693	676	693	693
14	PilF	K02656	AHA_1757	660	693	676	693	627	665	693	676	693	693
15	PilU	K02670	AHA_2739	1967	1967	1945	1945	1945	1906	1868	1951	2006	1929
16	PilQ	K02666	AHA_3190	660	693	676	693	627	665	693	676	693	693
17	PilP	K02665	AHA_3191	660	693	676	693	627	665	693	676	693	693
18	PilO	K02664	AHA_3192	660	693	676	693	627	665	693	676	693	693
19	PilN	K02663	AHA_3193	660	693	676	693	627	665	693	676	693	693
20	PilM	K02662	AHA_3194	660	693	676	693	627	665	693	676	693	693
21	PilT	K02669	AHA_3665	1967	1967	1945	1945	1945	1906	1868	1951	2006	1929
22	PilU	K02670	AHA_3666	1962	1962	1940	1940	1940	1901	1863	1946	2001	1924
23	PilZ	NA	AHA_3680	660	693	676	693	627	665	693	676	693	693
24	PilA	K02650	AHA_3868	126	165	161	126	154	159	182	159	126	182
25	PilB	K02652	AHA_3869	660	693	676	693	627	665	693	676	693	693
26	PilC	K02653	AHA_3870	2111	2073	2100	2078	2095	2134	2078	2084	2111	2073
27	PilD	K02654	AHA_3871	1441	1435	1391	1386	1463	1419	1452	1435	1380	1435

Table S4: (Contd.)

Sl. No.	T4P Component	KO No.	Locus tag	**A008N2**	**MX16A**	**B11**	**Brac6**	**Aer_ Brac14A**	**Aer_ Brac66**	**PartN-Ahydrophila-RM8376**	**FDAARGOS _916**	**71317**	**NEB724**
1	MshE	K12276	AHA_0392	2998	2998	2898	2931	2931	2931	2920	2920	2953	2200
2	FimT	K08084	AHA_0686	898	0	682	898	898	898	887	887	915	915
3	PilV	K02671	AHA_0687	665	0	499	688	688	688	693	693	665	665
4	PilW	K02672	AHA_0688	1829	0	1519	1901	1901	1901	1906	1906	1646	1646
5	PilX	K02673	AHA_0689	837	0	693	826	826	826	859	859	688	688
6	PilY	K02674	AHA_0690	5712	0	4582	5707	5707	5707	5784	5784	5657	5657
7	PilE	K02655	AHA_0691	693	0	547	688	688	688	671	671	699	699
8	PilE	K02655	AHA_0692	621	0	414	604	604	604	604	604	621	621
9	FimT	K08084	AHA_0693	909	0	340	865	865	865	893	893	909	909
10	NA	NA	AHA_0694	992	0	0	953	953	953	976	976	970	970
11	NA	NA	AHA_0695	693	0	547	688	688	688	671	671	699	699
12	NA	NA	AHA_0696	0	0	652	3386	3386	3386	3109	3109	3424	3424
13	PilZ	NA	AHA_0877	693	693	547	688	688	688	671	671	699	693
14	PilF	K02656	AHA_1757	693	693	547	688	688	688	671	671	699	693
15	PilU	K02670	AHA_2739	1956	1956	1707	1929	1929	1929	1917	1917	1956	1454
16	PilQ	K02666	AHA_3190	693	693	547	688	688	688	671	671	699	693
17	PilP	K02665	AHA_3191	693	693	547	688	688	688	671	671	699	693
18	PilO	K02664	AHA_3192	693	693	547	688	688	688	671	671	699	693
19	PilN	K02663	AHA_3193	693	693	547	688	688	688	671	671	699	693
20	PilM	K02662	AHA_3194	693	693	547	688	688	688	671	671	699	693
21	PilT	K02669	AHA_3665	1956	1956	1707	1929	1929	1929	1917	1917	1956	1454
22	PilU	K02670	AHA_3666	1951	1951	1702	1924	1924	1924	1912	1912	1951	1449
23	PilZ	NA	AHA_3680	693	693	547	688	688	688	671	671	699	693
24	PilA	K02650	AHA_3868	165	165	154	132	132	132	200	200	159	0
25	PilB	K02652	AHA_3869	693	693	547	688	688	688	671	671	699	693
26	PilC	K02653	AHA_3870	2073	2073	1773	2061	2061	2061	2061	2061	2001	1269
27	PilD	K02654	AHA_3871	1435	1435	1369	1463	1463	1463	1402	1402	1435	861

Table S4: (Contd.)

Sl. No.	T4P Component	KO No.	Locus tag	**WCHAH 045096**	**AL09-71**	**YL17**	**NJ-35**	**ML09-119**	**pc104A**	**J-1**	**4AK4**	**2359**	**3206**	**3924**
1	MshE	K12276	AHA_0392	2926	2898	0	2898	2898	2898	2898	2222	2933	2876	2909
2	FimT	K08084	AHA_0686	887	881	688	881	881	881	881	0	904	876	893
3	PilV	K02671	AHA_0687	676	704	510	704	704	704	704	0	671	680	665
4	PilW	K02672	AHA_0688	1906	1840	0	1840	1840	1840	1840	0	1834	1832	1840
5	PilX	K02673	AHA_0689	848	848	693	848	848	848	848	0	815	741	832
6	PilY	K02674	AHA_0690	5734	5712	4626	5712	5712	5712	5712	0	5734	5629	5541
7	PilE	K02655	AHA_0691	676	693	553	693	693	693	693	0	686	654	682
8	PilE	K02655	AHA_0692	610	643	420	643	643	643	643	0	621	621	632
9	FimT	K08084	AHA_0693	865	881	335	881	881	881	881	0	915	880	876
10	NA	NA	AHA_0694	1020	837	0	837	837	837	837	0	976	968	865
11	NA	NA	AHA_0695	676	693	553	693	693	693	693	0	686	654	682
12	NA	NA	AHA_0696	3435	3419	0	3419	3419	3419	3419	0	3419	3051	3624
13	PilZ	NA	AHA_0877	676	693	553	693	693	693	693	693	686	654	682
14	PilF	K02656	AHA_1757	676	693	553	693	693	693	693	693	686	654	682
15	PilU	K02670	AHA_2739	1973	1917	1768	1917	1917	1917	1917	1448	1989	1938	1851
16	PilQ	K02666	AHA_3190	676	693	553	693	693	693	693	693	686	654	682
17	PilP	K02665	AHA_3191	676	693	553	693	693	693	693	693	686	654	682
18	PilO	K02664	AHA_3192	676	693	553	693	693	693	693	693	686	654	682
19	PilN	K02663	AHA_3193	676	693	553	693	693	693	693	693	686	654	682
20	PilM	K02662	AHA_3194	676	693	553	693	693	693	693	693	686	654	682
21	PilT	K02669	AHA_3665	1973	1917	1768	1917	1917	1917	1917	1448	1989	1938	1851
22	PilU	K02670	AHA_3666	1968	1912	1763	1912	1912	1912	1912	1443	1984	1933	1846
23	PilZ	NA	AHA_3680	676	693	553	693	693	693	693	693	686	654	682
24	PilA	K02650	AHA_3868	148	171	154	171	171	171	171	0	159	159	128
25	PilB	K02652	AHA_3869	676	693	553	693	693	693	693	693	686	654	682
26	PilC	K02653	AHA_3870	2061	1995	1790	1995	1995	1995	1995	1236	2049	2111	2117
27	PilD	K02654	AHA_3871	1458	1408	1308	1408	1408	1408	1408	0	1434	1439	1419

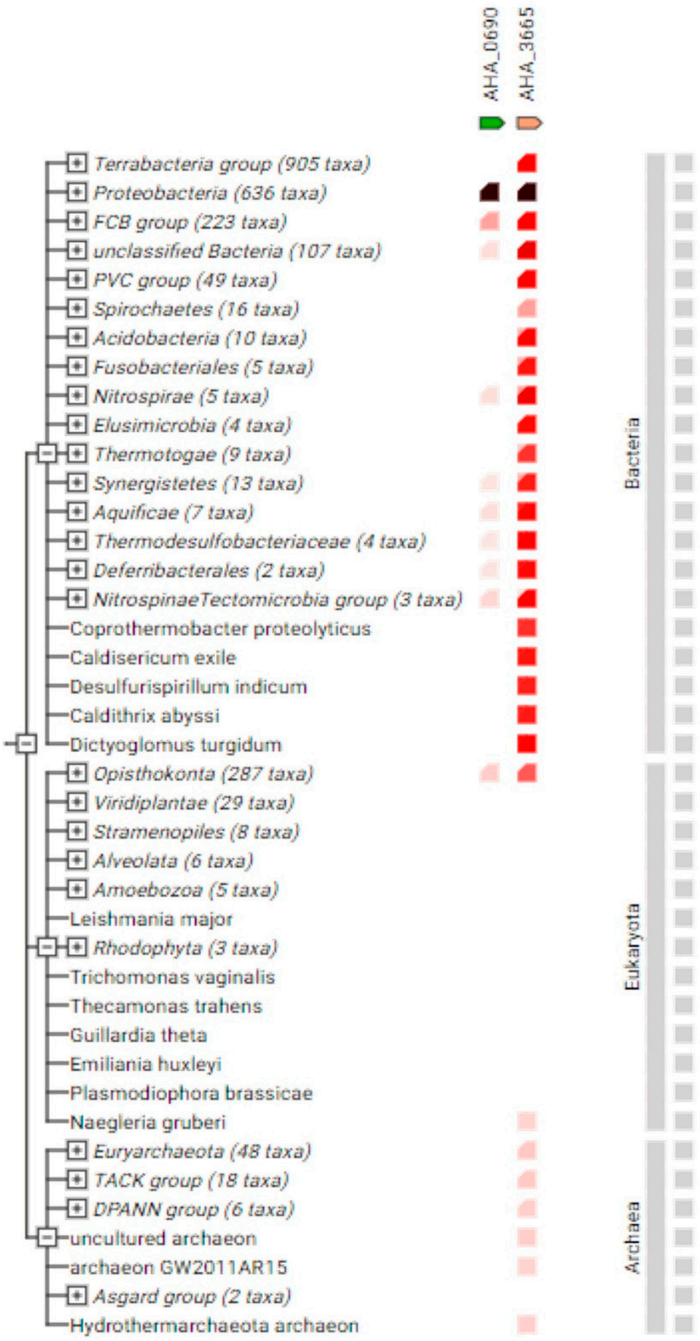

Figure S1: Cooccurrence of PilY and PilT across genomes indicating probable interaction between them.

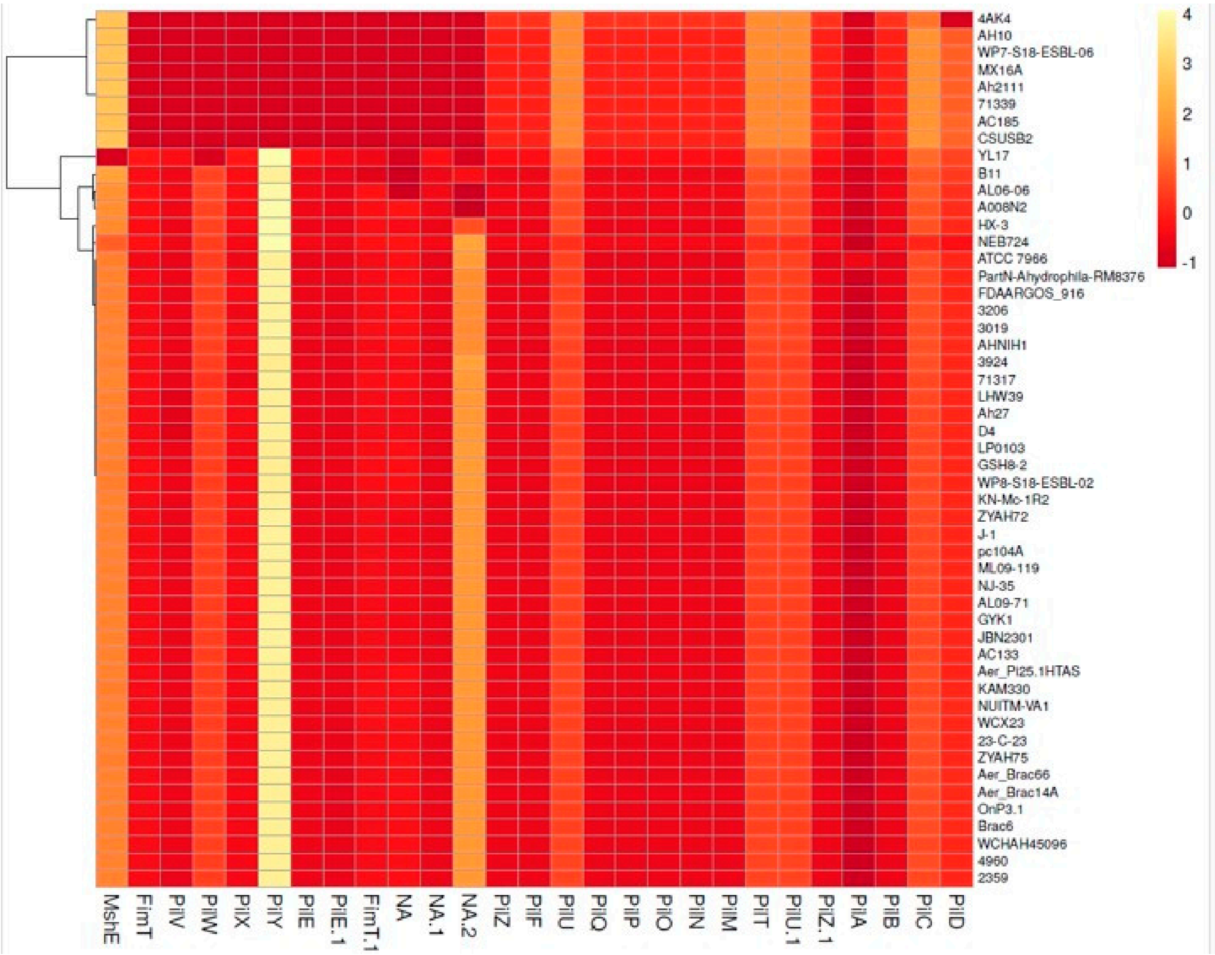

Figure S2: Hitmap showing distribution of T4P components within *51 Aeromonas hydrophila* genome.

References

1. Averhoff, B.; Kirchner, L.; Pfefferle, K.; Yaman, D. Natural transformation in Gram-negative bacteria thriving in extreme environments: from genes and genomes to proteins, structures and regulation. *Extremophiles* **2021**, 1-12.
2. Potapova, A.; Carreira, L.A.M.; Søgaard-Andersen, L. The small GTPase MglA together with the TPR domain protein SgmX stimulates type IV pili formation in M. xanthus. *Proceedings of the National Academy of Sciences* **2020**, *117*, 23859-23868.
3. Vahvelainen, N.; Bozkurt, E.; Maula, T.; Johansson, A.; Pöllänen, M.T.; Ihalin, R. Pilus PilA of the naturally competent HACEK group pathogen Aggregatibacter actinomycetemcomitans stimulates human leukocytes and interacts with both DNA and proinflammatory cytokines. *Microbial Pathogenesis* **2022**, *173*, 105843.
4. Dye, K.J.; Vogelaar, N.J.; Sobrado, P.; Yang, Z. High-throughput screen for inhibitors of the type IV pilus assembly ATPase PilB. *Msphere* **2021**, *6*, 10.1128/msphere. 00129-00121.
5. Rudel, T.; Scheuerpflug, I.; Meyer, T.F. Neisseria PilC protein identified as type-4 pilus tip-located adhesin. *Nature* **1995**, *373*, 357-359.
6. Strom, M.S.; Nunn, D.N.; Lory, S. [42] Posttranslational processing of type IV prepilin and homologs by PilD of Pseudomonas aeruginosa. *Methods in enzymology* **1994**, *235*, 527-540.
7. Darzins, A.; Russell, M.A. Molecular genetic analysis of type-4 pilus biogenesis and twitching motility using Pseudomonas aeruginosa as a model system–a review. *Gene* **1997**, *192*, 109-115.
8. Koo, J.; Tammam, S.; Ku, S.-Y.; Sampaleanu, L.M.; Burrows, L.L.; Howell, P.L. PilF is an outer membrane lipoprotein required for multimerization and localization of the Pseudomonas aeruginosa type IV pilus secretin. *Journal of bacteriology* **2008**, *190*, 6961-6969.
9. Liu, J.; Hu, L.; Xu, Z.; Tan, C.; Yuan, F.; Fu, S.; Cheng, H.; Chen, H.; Bei, W. Actinobacillus pleuropneumoniae two-component system QseB/QseC regulates the transcription of PilM, an important determinant of bacterial adherence and virulence. *Veterinary microbiology* **2015**, *177*, 184-192.
10. McCallum, M.; Tammam, S.; Little, D.J.; Robinson, H.; Koo, J.; Shah, M.; Calmettes, C.; Moraes, T.F.; Burrows, L.L.; Howell, P.L. PilN binding modulates the structure and binding partners of the Pseudomonas aeruginosa type IVa pilus protein PilM. *Journal of biological Chemistry* **2016**, *291*, 11003-11015.
11. Ozanic, M.; Marecic, V.; Knezevic, M.; Kelava, I.; Stojková, P.; Lindgren, L.; Bröms, J.E.; Sjöstedt, A.; Abu Kwaik, Y.; Santic, M. The type IV pili component PilO is a virulence determinant of Francisella novicida. *Plos one* **2022**, *17*, e0261938.
12. Balasingham, S.V.; Collins, R.F.; Assalkhou, R.; Homberset, H.v.; Frye, S.A.; Derrick, J.P.; Tønjum, T. Interactions between the lipoprotein PilP and the secretin PilQ in Neisseria meningitidis. *Journal of bacteriology* **2007**, *189*, 5716-5727.
13. Narulita, E.; Addy, H.S.; Kawasaki, T.; Fujie, M.; Yamada, T. The involvement of the PilQ secretin of type IV pili in phage infection in Ralstonia solanacearum. *Biochemical and biophysical research communications* **2016**, *469*, 868-872.
14. Hughes, H.Q.; Christman, N.D.; Dalia, T.N.; Ellison, C.K.; Dalia, A.B. The PilT retraction ATPase promotes both extension and retraction of the MSHA type IVa pilus in Vibrio cholerae. *PLoS Genetics* **2022**, *18*, e1010561.

15. Winther-Larsen, H.C.; Hegge, F.T.; Wolfgang, M.; Hayes, S.F.; Van Putten, J.P.; Koomey, M. Neisseria gonorrhoeae PilV, a type IV pilus-associated protein essential to human epithelial cell adherence. *Proceedings of the National Academy of Sciences* **2001**, *98*, 15276-15281.
16. Carbonnelle, E.; Hélaine, S.; Prouvensier, L.; Nassif, X.; Pelicic, V. Type IV pilus biogenesis in Neisseria meningitidis: PilW is involved in a step occurring after pilus assembly, essential for fibre stability and function. *Molecular microbiology* **2005**, *55*, 54-64.
17. Helaine, S.; Dyer, D.H.; Nassif, X.; Pelicic, V.; Forest, K.T. 3D structure/function analysis of PilX reveals how minor pilins can modulate the virulence properties of type IV pili. *Proceedings of the National Academy of Sciences* **2007**, *104*, 15888-15893.
18. Heiniger, R.W.; Winther-Larsen, H.C.; Pickles, R.J.; Koomey, M.; Wolfgang, M.C. Infection of human mucosal tissue by Pseudomonas aeruginosa requires sequential and mutually dependent virulence factors and a novel pilus-associated adhesin. *Cellular microbiology* **2010**, *12*, 1158-1173.
19. Guzzo, C.R.; Salinas, R.K.; Andrade, M.O.; Farah, C.S. PILZ protein structure and interactions with PILB and the FIMX EAL domain: implications for control of type IV pilus biogenesis. *Journal of molecular biology* **2009**, *393*, 848-866.
